# Acquired Idiopathic Generalized Anhidrosis (AIGA) and Its Complications: Implications for AIGA as an Autoimmune Disease

**DOI:** 10.3390/ijms22168389

**Published:** 2021-08-04

**Authors:** Reiko Kageyama, Tetsuya Honda, Yoshiki Tokura

**Affiliations:** 1 Department of Dermatology, Hamamatsu University School of Medicine, Hamamatsu 431-3192, Japan; hontetsu@hama-med.ac.jp; 2 Allergic Disease Research Center and Department of Dermatology, Chutoen General Medical Center, Kakegawa 436-8555, Japan; tokura@chutoen-hp.shizuoka.jp

**Keywords:** acquired idiopathic generalized anhidrosis, cholinergic urticaria, complication, autoimmune disorder

## Abstract

Acquired idiopathic generalized anhidrosis (AIGA) is a rare disorder in which systemic anhidrosis/hypohidrosis occurs without causative dermatological, metabolic or neurological disorder. Most cases of AIGA have been reported in Asia, especially in Japan, but there have been only a few reports in Europe and the United States. Severe AIGA may result in heatstroke and can reduce quality of life due to restriction of exercise and outdoor works. AIGA is often accompanied by cholinergic urticaria (CholU), and it is thought that AIGA and CholU with anhidrosis/hypohidrosis belong to the same spectrum of the disease. However, the pathophysiology of AIGA has not yet been clarified. Decreased expression of cholinergic receptor M3 on the epithelial cells of eccrine sweat glands is often accompanied by T cell infiltration around eccrine apparatus, suggesting an immunological mechanism of disordered perspiration. AIGA is occasionally associated with various complications indicative of autoimmune disorders. The association of autoimmune complications further suggests that AIGA is an autoimmune disorder. Studies on complications may lead to a better understanding of the pathophysiology of AIGA.

## 1. Introduction

Acquired idiopathic generalized anhidrosis (AIGA) is a rare disorder characterized by the sudden onset of systemic anhidrosis/hypohidrosis without directly causative dermatological, metabolic or neurological disorder [1]. Severe AIGA may result in heatstroke and can reduce quality of life due to restriction of exercise and outdoor works. A high frequency of the association with cholinergic urticaria (CholU) is noted in AIGA [1]. From the dermatological point of view, CholU is the primary nomenclature for this condition [2], and thus, AIGA has been historically reported as “CholU with anhidrosis” or “CholU with hypohidrosis” in the dermatological literature [3]. In the terminology for depressed perspiration, anhidrosis and hypohidrosis represent complete and incomplete lack of sweating, respectively. Currently, it is thought that AIGA and CholU with anhidrosis/hypohidrosis belong to the same spectrum of the disease [2], and not only neurologists but also dermatologists use AIGA in daily practice. Although AIGA is well known to show CholU and associated itch or even pain, the exact frequency of CholU in patients with AIGA has not been investigated. One of the reasons for the lack of research may be that the degree of CholU varies from case to case and endemic investigation on this issue is not easy.

Systemic administration of corticosteroids is recommended for the treatment of AIGA, and there is a report that other immunosuppressive agents such as cyclosporine are also effective [1,4,5]. Case reports that have been recently accumulated indicate that patients with AIGA have various complications or comorbidities, including autoimmune disorders. These complications may provide some implications to elucidate the pathology of AIGA, possibly because the patients developed AIGA and its complications in parallel via the similar mechanisms. This review article aims to show complications of AIGA in our and reported cases. The present review of literature further clarifies the significance of autoimmune diseases as important complications.

## 2. Epidemiology of AIGA and CholU with Anhidrosis/Hypohidrosis

More than 100 cases of AIGA have been documented worldwide, but no study has been conducted regarding large-scale epidemiology on AIGA, and thus, its exact prevalence is unknown [1,6]. While a vast majority of cases of AIGA have been reported in Asia, especially in Japan, only a few reports have been documented from Europe [6,7,8,9] and the United States [10,11,12]. Accordingly, when we investigated cases of CholU with anhidrosis/hypohidrosis in the literature (until 2010), 26 out of 29 cases were Japanese [3]. Thus, the ethnic differences in the frequency of AIGA definitely exists between Asian and Western populations.

AIGA can occur at any age, but evidence suggests that the prevalent onset age is often in the 20 s to 40 s. Considering that men of these ages are prone to sweat in their workplaces or upon exercise of daily life, they may easily realize disordered perspiration. There was a report of unusual onset of AIGA in a 2-year-old infant [13]. Provided that such a young patient suffers from AIGA, we should make a careful diagnosis for pediatric cases. In addition, the complaint of low perspiration is not clear in low-aged children under 6 years.

It is considered that AIGA is associated with three pathological conditions: sudomotor neuropathy, idiopathic pure sudomotor failure (IPSF), and sweat gland failure [1]. No attenuation of mental sweating, usually present in the palms/soles and axillae, is noted in IPSF [1]. Since IPSF is considered to account for about 90% of AIGA, it is regarded as a narrowly defined condition of AIGA. In particular, IPSF is a highly frequent condition in adolescent male patients. This is in accordance with the finding that AIGA is remarkably common in men, accounting for more than 80% of known cases [1]. AIGA is often accompanied by CholU, and therefore, AIGA and CholU with anhidrosis/hypohidrosis are considered to be within the same disease spectrum [2]. AIGA or IPSF is characterized by pain, which is also a main complication of CholU. On the basis of these epidemiological studies, the diagnostic usage of IPSF is less common than that of AIGA, and AIGA seems to be generally used for the condition.

## 3. Two Mechanisms of CholU and Relation to Anhidrosis/Hypohidrosis

**Figure 1:**
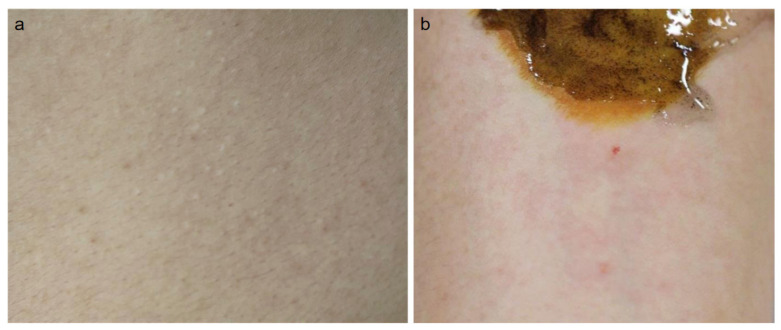
Clinical appearance of cholinergic urticaria (CholU) and acetylcholine (Ach) injection test. Pinpoint wheal of CholU (**a**). Satellite wheal around intradermal injection of Ach (**b**).

CholU manifests small, itchy and/or painful wheals occurring upon perspiration and mechanically involving acetylcholine (Figure 1). We proposed that CholU can be categorized into two major subtypes: acetylcholine-indirectly induced, sweat allergic type and acetylcholine-directly induced, depressed sweating type [2].

In the former type, acetylcholine (Ach) evokes perspiration, and some sweat antigen(s) leaking from the sweat ducts to the dermis may stimulate mast cells to release histamine. In this type, the ducts might be damaged or obstructed for sweat leakage, and patients frequently exhibit positive autologous sweat skin test, representing “sweat allergy”. This hypothesis has been supported by high frequency of positive autologous sweat skin test as well as positive Ach skin test in the patients [14]. In addition to this in vivo test, sweat allergy is also proven in vitro by basophil histamine release test and basophil activation test [14]. Concerning sweat antigens, *Malassezia globose*-derived substance, MGL_1304, was identified as a major allergen in human sweat for patients with atopic dermatitis and CholU [15]. These patients have high levels of serum IgE to purified MGL_1304 from sweat [15], suggesting that the *Malassezia*-derived substance plays a causative role for the diseases.

On the other hand, the latter Ach-mast cell directly interacting type, typically seen as “CholU with anhidrosis and/or hypohidrosis”, eccrine sweat gland epithelial cells lack CHRM3 expression. The expression of CHRM3 is completely absent in the anhidrotic areas and only slightly expressed in the hypohidrotic areas. In the hypohidrotic area, where pinpoint wheal occurs, it is hypothesized that released Ach cannot be completely trapped by CHRM3 of eccrine glands and overflows to the adjacent mast cells, leading to wheal formation [3]. Thus, sweat allergy is not a requirement in this depressed sweating type. Although some additional complications, such as angioedema, anaphylaxis, and cold urticaria, have been documented, these two types represent the modes of action of Ach in this enigmatic urticaria.

## 4. Immunological Findings in AIGA

Although there is no sufficiently high level of evidence for its effectiveness, systemic corticosteroid therapy is recommended for the early stages of AIGA or CholU with anhidrosis/hypohidrosis [1,16]. This suggests that AIGA and related CholU are mediated via immunological and inflammatory mechanisms. In fact, decreased expression of CHRM3 on the epithelial cells of eccrine sweat glands was found in AIGA or CholU with anhidrosis/hypohidrosis.

It is known that lymphocytes infiltrate around eccrine glands in some patients with CholU. Historically, different lines of studies have suggested that two pathogenic consequences may follow the lymphocytic infiltration. The first consequence is the damage or obstruction of sweat ducts and the resultant dermal leakage of sweat. In the second one, the CHRM3 expression on sweat gland epithelial cells is decreased by the infiltrating lymphocytes. The first and second outcomes are related to the Ach-indirectly induced, sweat allergic type, and the Ach-directly induced, depressed sweating type, respectively. It appears that each of these two consequences occurs almost exclusively. We consider that the second one is more closely associated with lymphocytic infiltration [17]. The infiltrate consists of both CD4^+^ and CD8^+^ T cells, and as assessed by the expression of CXCR3 and CCR4, both Th1/Tc1 and Th2/Tc2 subpopulations are identifiable [17].

**Figure 2:**
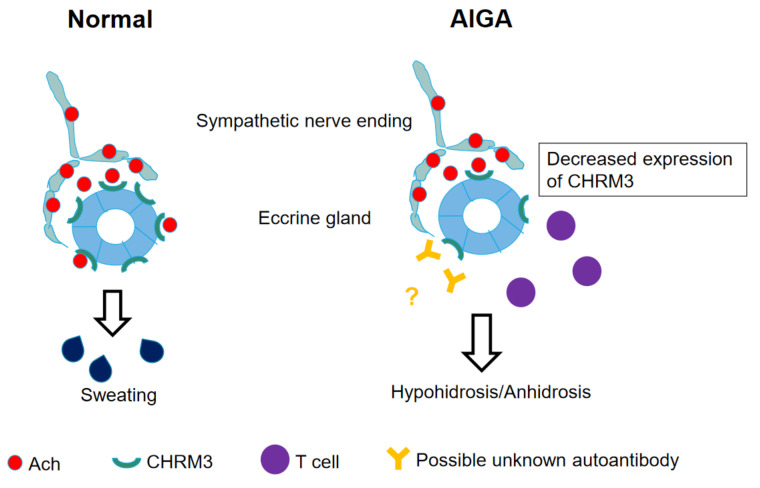
Summary of the immune mechanisms underlying AIGA.

Although the mechanism underlying the decreased expression of CHRM3 by CD4^+^ and/or CD8^+^ T cells remains unknown, T cells chemoattracted by CCL2/MCP-1, CCL5/RANTES, CCL17/TARC are possibly responsible for the reduced CHRM3 expression [17]. In addition to the T cell-mediated receptor down-modulation theory, some autoantibodies directed against CHRM3 might affect the receptor-mediated signaling (Figure 2). These different lines of evidence prompt us to investigate the autoimmune mechanism underlying AIGA.

## 5. Decreased Expression of CHRM3 in AIGA

The mechanisms underlying AIGA remain to be clarified. In AIGA patients, decreased expression of CHRM3 has often been observed in sweat gland epithelial cells [17]. In addition to the impaired expression of CHRM3, other abnormalities have been proposed in AIGA, such as decreased expression of acetylcholine esterase [17]. There are reports that serum carcinoembryonic antigen (CEA) levels are increased in AIGA patients and CEA may serve as a clinical marker for AIGA activity [18,19]. The relationship between aquaporin-5 and AIGA has also been reported and is another issue to be further elucidated [20].

In AIGA, the disordered perspiration is not homogenous over the skin. The skin surface of depressed sweating can be divided into the two areas, anhidrosis and hypohidrosis, as assessed by iodine-starch test [3]. The expression of CHRM3 is completely absent in the anhidrotic areas, while it is incompletely decreased in the hypohidrotic areas. Typically, the predilection sites of anhidrosis include the four extremities (especially the distal parts), while the chest, abdomen and back are hypohidrotic areas. Sweating is usually retained in the palms, soles and axillae. When the patients are successfully treated with corticosteroid pulse therapy, the anhidrotic area is gradually diminished and replaced by the hypohidrotic area, which is then further improved to normal sweating area [16]. The number of lymphocytes infiltrating in eccrine glands inversely correlated with the expression levels of CHRM3 [17,21]. Therefore, it is possible that CHRM3 expression on sweat gland epithelial cells is decreased by the infiltrating lymphocytes.

Ach is historically known to induce degranulation and subsequent histamine release in mast cells [22,23]. Since mast cells infiltrate in the vicinity of sympathetic nerves, these in vitro findings strongly suggest that Ach stimulate mast cells to secrete histamine in certain pathogenic settings. A careful observation reveals that wheals occur on the hypohidrotic areas, but not on the anhidrotic areas. Therefore, CholU tends to take place on the perspiration-recovering area [16]. We are attempting to speculate that, in the hypohidrotic area, Ach released from nerves is not completely trapped by cholinergic receptors of eccrine glands and overflows to the adjacent mast cells, leading to wheal formation. In this scenario, mast cells can produce histamine in response to Ach via CHRM3, whose expression is low but still functional in the hypohidrotic area.

## 6. Autoimmune Diseases as Representative Complications or Comorbidities of AIGA

Various complications or comorbidities have been reported in AIGA. Atopic dermatitis and asthma were well documented, but other diseases presumably caused via autoimmune mechanisms are also associated with AIGA (Table 1). Such diseases are described below with the pathogenic similarities between the complications and AIGA per se.

### 6.1. Central Diabetes Insipidus

Central diabetes insipidus is characterized by hypotonic polyuria due to impairment of vasopressin (AVP) secretion from the posterior pituitary [24]. This disorder may be familial, idiopathic, or secondary [25]. Secondary diabetes insipidus is caused by tumors, infections, trauma, or other processes that damage the hypothalamic-neurohypophysial system. Idiopathic diabetes insipidus is characterized by selective hypofunction of the hypothalamic-neurohypophysial system. Central diabetes insipidus can be accompanied by decreased perspiration. Investigation of the reported cases indicates that there are two types of anhidrosis/hypohidrosis associated with central diabetes insipidus. In one type, decreased perspiration may secondarily occur as a consequence of diabetes insipidus [26], and in the other type, both diabetes insipidus and decreased perspiration may be induced by pathologically similar autoimmune mechanisms [27]. The condition can be caused by lymphocytic infundibuloneurohypophysitis, a disorder estimated as an autoimmune disorder [25], suggesting the latter autoimmune type.

**Figure 3:**
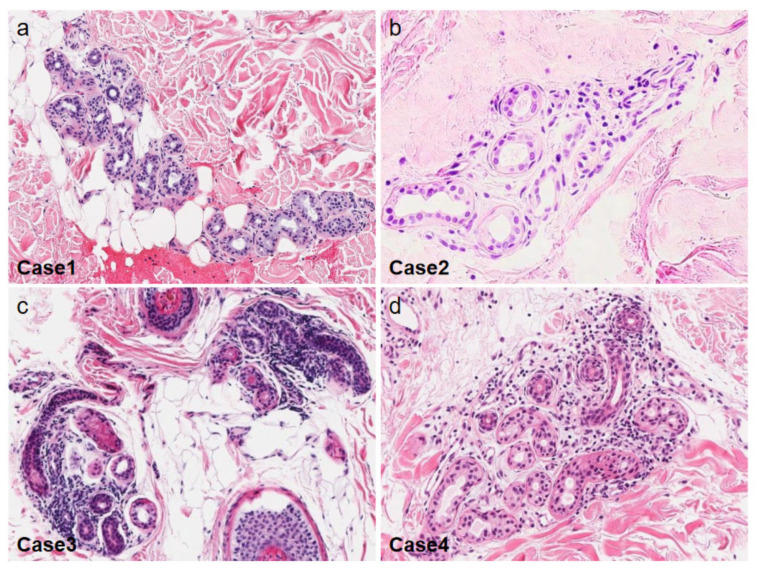
Skin histopathology of the anhidrotic area in our cases. Mild to moderate lymphocyte infiltration around the sweat glands is observed in Cases 1, 3, and 4 (**a**,**c**,**d,** original magnification ×40, ×100 and ×100 respectively), but scarce in Case 2 (**b,** original magnification ×200) (Hematoxylin-Eosin staining).

Our patient developed AIGA almost at the same time as the occurrence of central diabetes insipidus [28]. Although his central diabetes insipidus improved with oral administration of desmopressin acetate hydrate, the hypohidrosis had continued, and his histopathological study in the anhidrotic area showed mild lymphocyte infiltration around the sweat glands and mild sweat glands atrophy (Figure 3a). Meanwhile, the patient’s hypohidrosis was improved by corticosteroid pulse therapy. This mode of therapeutic action indicates that both diabetes insipidus and the decreased perspiration in our case stem from autoimmunity.

There is another case report that central diabetes insipidus was associated with AIGA, and the patient developed central diabetes insipidus 3 years after occurrence of AIGA [27]. In accordance with our case, the patient’s hypohidrosis was improved by corticosteroid pulse therapy, while his central diabetes insipidus was alleviated by administration of desmopressin acetate hydrate.

These two cases suggest that AIGA associated with central diabetes insipidus can be improved by only corticosteroid pulse therapy, but not by desmopressin acetate hydrate. In another case, however, administration of desmopressin acetate hydrate improved hypohidrosis as well as diabetes insipidus [26], supporting the notion that anhidrosis/hypohidrosis could occur as a secondary condition of central diabetes insipidus.

The therapeutic effectiveness of desmopressin acetate hydrate on anhidrosis/hypohidrosis is different among these cases. However, at least two cases provide evidence that AIGA is not a secondary condition of diabetes insipidus, but it occurs as an immunological disorder in parallel with central diabetes insipidus. Among various autoimmune diseases, diabetes insipidus has been paid attention as a complication of AIGA. This seems to be derived from the notion that the disordered perspiration is easily estimated as a related condition of diabetes insipidus. Although this mechanism may take place, the immunological mechanisms also contribute to both diseases.

### 6.2. Rheumatoid Arthritis

There is one report of a patient with rheumatoid arthritis who developed AIGA [29]. A 67-year-old man visited a hospital with a 2-month history of hypohidrosis. He had an 8-year history of rheumatoid arthritis and had been treated with methotrexate, abatacept, and insulin. He had hypohidrosis over his body except for his axillae. Histopathological studies in his anhidrotic area showed a poor lymphocytic infiltrate around the sweat glands and a decreased number of sweat glands. He was diagnosed as having AIGA and his hypohidrosis was improved immediately by corticosteroid pulse therapy.

It is interesting to investigate the findings in this case. First, rheumatoid arthritis is a disorder in which both T cells and antibodies are involved. This raises the possibility that his hypohidrosis could be induced by either of immune reactions or both. Second, he was treated with immunosuppressants, which might result in depressed infiltration of lymphocytes. In any case, the effectiveness of corticosteroid pulse therapy suggests that the patient’s hypohidrosis was AIGA. Evaluation of rheumatoid arthritis as a related complication or merely incidental condition is a future issue to be clarified with additional cases.

### 6.3. Crohn’s Disease

This inflammatory bowel disease was reported as a comorbidity of AIGA [30]. A 41-year-old man with Crohn’s disease developed hypohidrosis 3 months ago. He had hypohidrosis almost all over the skin except for the axillae and right elbow. Histopathological studies of his anhidrotic skin showed no abnormal findings. He was diagnosed as having AIGA. His hypohidrosis was improved by corticosteroid pulse therapy and no relapse was observed.

In Crohn’s disease, microbial dysbiosis results in uncontrolled intestinal inflammation, and the intestinal barrier formed by intestinal epithelial cells and the innate immune system are also compromised [31,32]. Acquired and innate T cell responses to a subset of commensal enteric bacteria operate in Crohn’s disease. AIGA might share such T cell responses with Crohn’s disease.

### 6.4. Alopecia Areata

AIGA was accompanied by alopecia areata [33]. A 43-year-old man visited a hospital with a one-year-history of hypohidrosis. He had hypohidrosis almost all over his body. Histopathological studies in his anhidrotic area showed mild lymphocyte infiltration around the sweat glands. After diagnosis of AIGA, corticosteroid pulse therapy was initiated. His hypohidrosis was improved by the treatment, but it was relapsed. He developed alopecia areata one year after initiation of the therapy. His hypohidrosis was relapsed in winter season and his alopecia areata also progressed in parallel with exacerbation of hypohidrosis.

Alopecia areata is regarded as a tissue-specific and cell-mediated autoimmune disorder, in which the collapse of hair follicle immune privilege plays a key role [34,35]. Antibodies against multiple components of hair follicles almost exclusively attack in the anagen phase, where melanogenesis takes place, suggesting involvement of melanogenesis-associated autoantigens as a target epitope. In the reported cases of AIGA, alopecia areata progressed in parallel with worsened hypohidrosis, suggesting autoimmune mechanisms underlying these two diseases.

### 6.5. Vitiligo Vulgaris and Vogt-Koyanagi-Harada Disease

These diseases have been reported as comorbidities of AIGA [36,37]. Vitiligo vulgaris is an acquired disease in which melanocytes in the skin are decreased or disappear. It is thought to be caused mainly by autoimmune loss of melanocytes from the involved areas [38,39,40]. Vitiligo vulgaris is frequently associated with autoimmune comorbidities and diseases, such as autoimmune thyroiditis, rheumatoid arthritis, type 1 diabetes, psoriasis, systemic lupus erythematosus, and alopecia areata, suggesting that AIGA shares the autoimmune mechanisms with these representative diseases. It is reported that both anti-thyroid antibodies and several melanocyte-related autoantibodies are elevated in the sera of patients with vitiligo vulgaris [41,42].

### 6.6. Lichen Planus

Lichen planus, which is one of the inflammatory diseases with dyskeratosis of the epidermis, was reported as a complication of AIGA [43]. In most of the affected individuals, the exact cause of lichen planus is unclear, except for medicine as a causative reagent. In lichen planus, some triggers stimulate the immune system to attack epidermal keratinocytes or mucous membranes. In this sense, lichen planus is an autoimmune disorder and associated with various immune-related diseases [44,45].

## 7. AIGA-Associated Autoimmune Diseases in Our Cases and Literature

**Figure 4:**
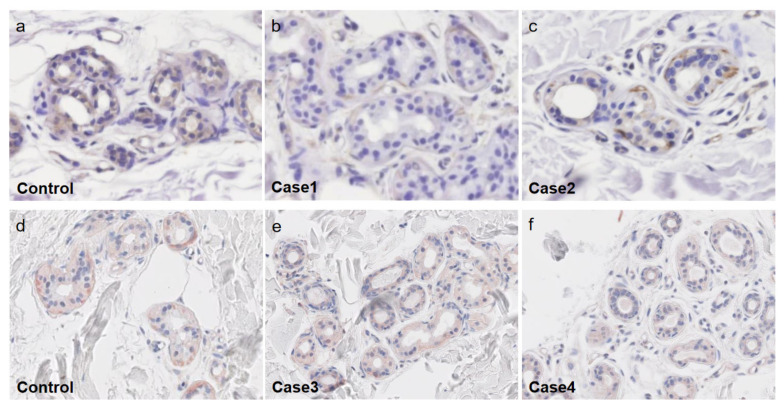
Immunohistochemical staining of cholinergic receptor M3 (CHRM3) at the anhidrotic area in our cases. The upper and lower columns were stained independently. Decreased expression of the CHRM3 (brown signals) is observed in sweat gland epithelial cells (**b**,**c**,**e**,**f,** original magnification ×200, ×200, ×100 and ×100 respectively) compared with control (**a**,**d,** original magnification ×200 and ×100 respectively).

**Figure 5:**
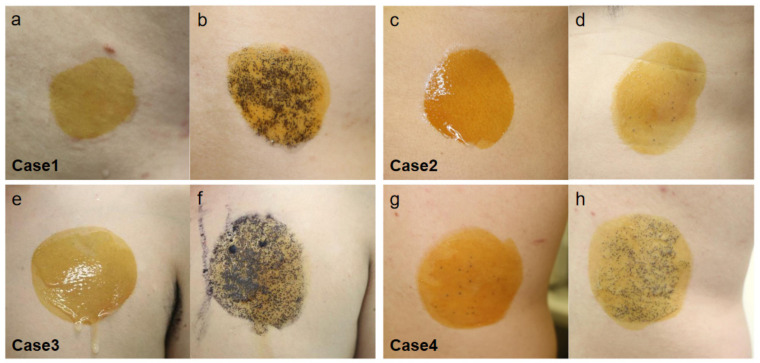
Thermoregulatory sweat test by Minor method using the iodine-starch reaction in our cases. No sweat is observed before treatment in all cases (**a**,**c**,**e**,**g**). Sweating is observed after corticosteroid pulse therapy in Cases 1, 3, and 4 (**b**,**f**,**h**), but not in Case 2 (**d**).

We experienced four AIGA cases with complications, including central diabetes insipidus [28], hypogammaglobulinemia [46], Basedow’s disease, and pulmonary sarcoidosis with psoriasis vulgaris (Table 1). In all of our cases, CHRM3 expression was decreased in the eccrine gland epithelial cells (Figure 4). Histopathological studies in the anhidrotic area showed mild to moderate lymphocyte infiltration around the sweat glands in Cases 1, 3, and 4 (Figure 3). Corticosteroid pulse therapy was effective for the reduced perspiration in three out of four cases (Figure 5).

The complications in our cases were characterized by presumed autoimmune mechanisms, supporting the notion that patients with AIGA have a high frequency of autoimmune disorders. Accordingly, histopathological examination of the anhidrotic/hypohidrotic skin in AIGA showed infiltration of CD3^+^ T cells around the eccrine sweat glands, suggesting that AIGA is induced by autoimmune T cell reactions. In this context, there was reported an interesting case of metastatic melanoma in which the patient developed AIGA after treatment with an immune checkpoint inhibitor [47]. As complications, the patient had vitiligo, gastritis, and hepatitis as adverse immune events and subsequently developed AIGA. The skin biopsy specimen taken from the anhidrotic area demonstrated a marked peri-eccrine lymphocyte infiltration. This patient’s sweating disorder was improved by systemic corticosteroid administration. This observation supports the concept that the patient’s anhidrosis/hypohidrosis is a lymphocyte-mediated adverse immune event of the immunotherapy. It is also suggested that AIGA may be induced by autoimmune T cell reactions.

It has been reported that anti-CHRM3 autoantibodies in the sera were detected in one of 12 AIGA patients [48]. In addition to autoreactive T cells, therefore, autoantibodies to the acetylcholine receptor might be responsible for the depressed perspiration in these autoimmune diseases. Peri-glandular Lymphocytic infiltrates can be found in AIGA, but not in all cases, raising a possibility that antibodies play a role in lymphocyte-paucity cases. There is an idea that lymphocytic infiltrates are likely to be found in the early onset of AIGA [49], suggesting an alternative possibility that lymphocytes disappear in the course.

## 8. Conclusions

The association of autoimmune complications implies that AIGA is an autoimmune disorder. It has been reported that both CD4^+^ and CD8^+^ T cells infiltrate around eccrine gland epithelial cells in AIGA and CholU with anhidrosis/hypohidrosis [17]. Further T cell subsets have not been investigated in AIGA. The complications provide insights for the subpopulations of T cells. Th17 cells are involved in the pathology of Crohn’s disease and rheumatoid arthritis [32,50]. In addition, skin resident memory T cells induce psoriasis and vitiligo [51]. In addition to Th and Tc cells, these T cell populations are possible candidates for the pathology.

Based on the autoimmune mechanism, corticosteroid therapy, and in particular corticosteroid pulse therapy, is recommended as the primary treatment for AIGA [1], although it does not improve all cases. It has been reported that younger age and delay from the onset to the treatment are negative factors for the effectiveness of AIGA treatment [4]. Seasonal factors have a significant effect on both treatment and recurrence for AIGA in Japan [4]. The effectiveness of oral administration of cyclosporine and Chinese herbal medicine has been reported in a limited number of the patients [5]. Studies on complications may lead to a better understanding of the pathophysiology of AIGA. Future studies are required to elucidate the pathophysiology of AIGA.

## Figures and Tables

**Table 1:** Summary of our AIGA cases and other reported AIGA cases with complications or comorbidities that are considered to be autoimmune diseases or diseases with presumed autoimmune mechanisms.

Cases	Age	Sex	Complication or Comorbidity	Period from AIGA Onset to the First Visit	Lymphocyte Infiltration around the Sweat Glands in Non-Sweat Area	CHRM3 Expression	Clinical Outcome with Steroid Pulse Therapy
Our case1	55	M	Central diabetes insipidus	15 years	Mild Mild sweat glands atrophy	Decreased	Improved sweating
Our case2	41	M	Hypogammaglobulinemia	10 years	Almost none Mild sweat glands atrophy	Decreased	No significant improvement in sweating
Our case3	42	M	Basedow’s disease	2 years	Mild to moderate	Decreased	Improved sweating
Our case4	32	M	Pulmonary sarcoidosis, Psoriasis vulgaris	3 months	Mild	Decreased	Improved sweating
Shimoda et al., 2019	43	M	Alopecia areata	1 year	Mild	N/A	Improved sweating
Kurachi et al., 2018	67	M	Rheumatoid arthritis	2 months	Almost none	N/A	Improved sweating
Gangadharan et al., 2015	47	M	Lichen planus	3 years	None Absence of sweat glands	N/A	Improved sweating
Saito et al., 2015	50	M	Vitiligo vulgaris	5 months	Moderate	N/A	Improved sweating
Sakaguchi et al., 2013	45	M	Vogt-Koyanagi-Harada disease	20 years	Mild Sweat glands atrophy	N/A	Improved sweating
Ohshima et al., 2011	41	M	Crohn’s disease	3 months	None	N/A	Improved sweating
Asahina et al., 2004	25	M	Central diabetes insipidus	19 months	None	N/A	Improved sweating
